# Three-dimensional reconstruction of anomalous eutectic in laser remelted Ni-30 wt.% Sn alloy

**DOI:** 10.1088/1468-6996/16/6/065007

**Published:** 2015-12-14

**Authors:** Yong-Qing Cao, Xin Lin, Zhi-Tai Wang, Li-Lin Wang, Meng-Hua Song, Hai-Ou Yang, Wei-Dong Huang

**Affiliations:** 1 State Key Laboratory of Solidification Processing, Northwestern Polytechnical University, 127 West Youyi Road, Xi’an, Shaanxi 710072, People’s Republic of China; 2 School of Materials Science and Engineering, Xi’an University of Technology, 5 South Jinhua Road, Xi’an, Shaanxi 710048, People’s Republic of China

**Keywords:** anomalous eutectic, laser remelting, Ni–Sn alloy, 3D reconstruction

## Abstract

Laser remelting has been performed on Ni-30 wt.% Sn hypoeutectic alloy. An anomalous eutectic formed at the bottom of the molten pool when the sample was remelted thoroughly. 3D morphologies of the *α*-Ni and Ni_3_Sn phases in the anomalous eutectic region were obtained and investigated using serial sectioning reconstruction technology. It is found that the Ni_3_Sn phase has a continuous interconnected network structure and the *α*-Ni phase is distributed as separate particles in the anomalous eutectic, which is consistent with the electron backscatter diffraction pattern examinations. The *α*-Ni particles in the anomalous eutectic are supersaturated with Sn element as compared with the equilibrium phase diagram. Meanwhile, small wavy lamella eutectics coexist with anomalous eutectics. The Trivedi–Magnin–Kurz model was used to estimate undercooling with lamellar spacing. The results suggest that the critical undercooling found in undercooling solidification is not a sufficient condition for anomalous eutectic formation. Besides, *α*-Ni particles in the anomalous eutectic do not exhibit a completely random misorientation and some neighboring *α*-Ni particles have the same orientation. It is shown that both the coupled and decoupled growth of the eutectic two phases can generate the *α*-Ni + Ni_3_Sn anomalous eutectic structure.

## 1. Introduction

Microstructure evolution during solidification is important in material research [1]. Eutectic solidification is an important solid–liquid phase transformation process. In recent years, microstructure evolution in eutectic alloys under rapid solidification has been widely studied. In some eutectic alloys, when the alloy melts are undercooled exceeding a critical undercooling, anomalous eutectics are yielded with a completely different morphology to regular lamellar or rod eutectics [2]. Experiments have shown that the formation of anomalous eutectics is a common phenomenon in the solidification of undercooled eutectic alloy melts and that it exists in many eutectic alloy systems, such as Ag–Cu [3, 4], Co–Sb [5, 6], Co–Ge [7], Ni–Sb [8], Co–Sn [9, 10] and Ni–Sn [11–17] eutectic alloys. Although there have been many reports on anomalous eutectics, many explanations and controversies still exist regarding the formation mechanism of anomalous eutectics.

Most of the previous studies have focused on the anomalous eutectic formed in the rapid solidification of undercooled alloy melts; however, it is interesting to note that the critical undercoolings obtained for the appearance of an anomalous eutectic in the same alloy system generally differs between studies. Taking Ni–Sn eutectic alloy as an example, the critical undercooling for the *α*-Ni + Ni_3_Sn anomalous eutectic were measured to be 100 K by Wei *et al* [13], 50 K by Li *et al* [14], 20 K by Li *et al* [15] and 10 K by Xing *et al* [16]. The difference between them is very large. It should be indicated that there exists a variation in microstructure across the undercooling solidified samples. Among these previous works, Li *et al* [15] described the microstructural evolution from the surface to the center of the samples in more detail. They found that the anomalous eutectics appear in the center of the coupled eutectic dendrite when the undercooling is larger than 20 K. This means that the critical undercooling of 20 K for the formation of an anomalous eutectic in the undercooling solidification of Ni–Sn eutectic alloy could be reasonable.

The solid/liquid (S/L) interface velocity is also generally recognized as an important factor in understanding the formation of anomalous eutectics. Li *et al* [15] calculated the variation of the growth velocities of *α*-Ni + Ni_3_Sn eutectic dendrite and *α*-Ni dendrite with undercooling in the solidification of Ni-18.7at.%Sn melt. The two velocity curves intersect at an undercooling of 135 K, below which the eutectic dendrite grows faster than the *α*-Ni dendrite, while above which the *α*-Ni dendrite grows faster, which was in good agreement with their experimental measurement of 130 K. They indicated that the coupled lamella eutectic dendritic growth occurred below the undercooling of 130 K and the decoupled single-phase growth occurred at the higher undercoolings, suggesting that the disintegration of both lamellar eutectics and single phase dendrites can result in anomalous eutectics. Yang *et al* [17] used a high speed camera to measure the crystal growth velocity of Ni-18.7at.%Sn eutectic alloy. The measured crystal growth velocities increased monotonically with undercooling. However, a sudden rise in the crystal growth velocities occured for an undercooling of about 160 K. They interpreted such a phenomenon to be the sign of a transition in crystal growth mode from the coupled growth of eutectic dendrites to the rapid uncoupled growth of single-phase *α*-Ni dendrites. Previous works have used *in situ* high speed imaging technology to monitor the solid–liquid interface in the solidification of undercooled melts. However, Li *et al* [14] pointed out that the interface velocity could not be measured exactly due to copious nucleation in the Ni-18.7at.%Sn eutectic melt regardless of the melt undercooling in the unconstrained solidification. Recently, laser solid forming and laser surface remelting, as another kind of rapid solidification technology, were applied to investigate the formation of anomalous eutectics. When compared with other high undercooling solidification techniques, the S/L interface moving velocity in the laser processing process can be controlled and measured exactly. Lin *et al* [18, 19] found that the anomalous eutectic formed during the laser solid forming of a graded Ti-xRene88DT alloy and Ti6Al4V-xRene88DT alloy. Recently, Wang *et al* [20] observed an anomalous eutectic during laser remelting of Ni-33 wt. %Sn hypereutectic alloy when the sample was melted thoroughly using a low scanning velocity.

It should be emphasized that the anomalous eutectic is generally classified by its morphology. The morphology of the anomalous eutectic is usually examined based on two-dimensional (2D) images obtained by an optical microscope (OM) or a scanning electron microscope (SEM). However, some important microstructure characteristics including spatial geometry, spatial distribution and connectivity of the eutectic phases cannot be fully demonstrated through the observation in 2D planar sections. Contieri *et al* [21] used the serial sectioning technique to reconstruct the microstructure of a Nb–Al–Ni ternary eutectic obtained by directional solidification. Their observation showed that there was very distinct difference between the three-dimensional (3D) eutectic microstructure characteristic and the understanding based on the 2D metallograph. From the 2D images, it is difficult to distinguish the spatial characteristics between the different eutectic phases, which is very important in understanding the growth mechanism of the anomalous eutectic.

In this paper, Ni-30 wt.% Sn hypoeutectic alloy was remelted thoroughly using a low scanning velocity by a laser beam in order to obtain a Ni–Sn anomalous eutectic. Through successively polishing serial sections and taking photos using an optical microscope, the 3D morphology of the anomalous eutectic was reconstructed. Then the spatial distribution and connectivity of the eutectic phases were investigated. The overall orientation of the eutectic phases in the anomalous eutectic zone was also characterized by the electron backscatter diffraction pattern (EBSD). Finally, the formation condition of the Ni–Sn anomalous eutectic was discussed.

## 2. Experimental procedures

**Figure 1.**
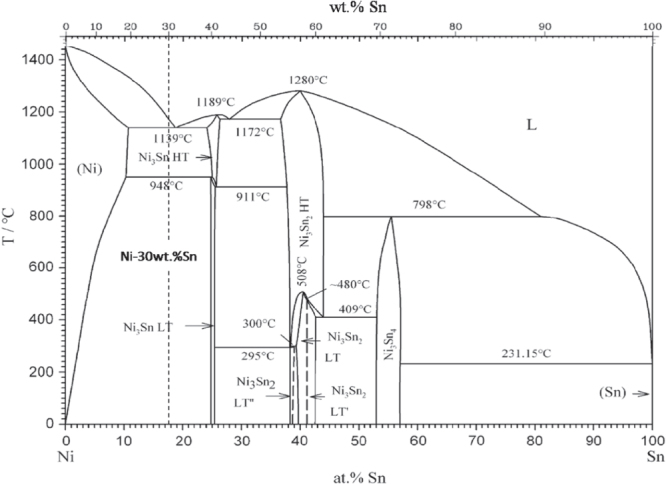
The Ni–Sn phase diagram modified from Schmetterer *et al* [22].

A Ni-30 wt.% Sn hypoeutectic alloy ingot, whose phase diagram is shown in figure 1, was prepared from high purity nickel (>99.99 wt.%) and tin (>99.999 wt.%) in the electric arc melting furnace with a water-cooled copper crucible. The ingot was remelted at least three times to homogenize the alloy composition. Specimens with a thickness of 2 mm were cut from the ingot. All specimens were polished and cleaned thoroughly by acetone to ensure a similar surface condition before laser surface remelting. The laser remelting experiment was carried out using a continuous wave CO_2_ laser with a nominal power of 2200 W [20]. The diameter of the laser beam was set to be 2 mm and the scanning speed was 0.1 mm s^−1^. In order to reduce heating of the specimens, they were placed on a copper plate. Ni–Sn alloys were remelted in an argon shielded glove box to prevent oxidation during the laser surface remelting.

The remelted specimens were sectioned perpendicular to the laser trace. To prepare the samples for optical microscopy observation, the samples were first ground with SiC paper, then polished using the colloidal silica solution (OP-S). Because the OP-S solution has the effect of chemical etching, the samples can be observed directly after polishing without further etching. Optical micrographs were taken by an OLYMPUS-GX71 optical microscope. The microstructure and composition distribution were further characterized using a TESCAN VEGAII LMH scanning electron microscope (SEM) with an energy dispersive spectrometry (EDS) analysis facility. The grain orientation of the anomalous eutectic was examined by electron backscattered diffraction (EBSD) analysis. Before EBSD analysis, electrolytic polishing was performed after the mechanical polishing of the sample, which was used to eliminate the residual deformation at the sample surface.

For 3D reconstruction of the microstructure, serial sectioning of the samples was carried out. In order to ensure subsequent section alignment, three random regions on the sample surface were chosen and marked by Vickers hardness indentations using a Duramin-A300 microhardness tester. The removal of material between the adjacent sections was performed by a Struers semi-automatic polisher, which applied a constant pressure to the sample on the polishing cloth for a fixed amount of time, and provided a reliable material removal rate of 0.59 *μ*m thickness per layer. After alignment in an optical microscope, photographs of each layer through the hardness indentations were taken. Mimics software was used to reconstruct the 3D morphology of the microstructure. The reconstruction region is about 97.1 *μ*m × 90.2 *μ*m × 21.8 *μ*m.

## 3. Results and discussion

### 3.1. As-remelted microstructure

**Figure 2.**
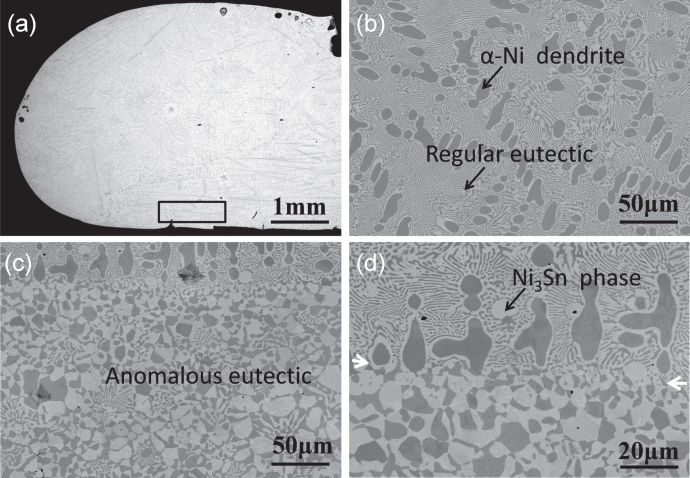
Backscattered electron images of typical macrostructure and microstructure of laser remelted Ni-30 wt.% Sn alloy: (a) macro morphology of the specimen; (b) typical structure in the specimen; (c) anomalous eutectic at the bottom of the specimen; (d) the transition zone of typical structure and anomalous eutectic. (*P* = 2200 W, *V* = 0.1 mm s^−1^).

Figure 2 shows the SEM backscattered electron images of the typical macrostructure and microstructure of laser remelted Ni-30 wt.% Sn hypoeutectic alloy. The black area is the *α*-Ni phase and the white area is the Ni_3_Sn phase. The overall macrostructure of the as-remelted specimen is shown in figure 2(a). The left edge of the specimen has a relatively smooth oval shape. This is due to the fact that the specimen was remelted thoroughly and then resolidified in an ellipsoid due to surface tension. The microstructure of the remelted specimen is mainly composed of the coarse primary *α*-Ni dendrite and the fine regular *α*-Ni + Ni_3_Sn lamellar and rod eutectic in the interdendritic region, as shown in figure 2(b). The overall microstructure is distributed uniformly except for the anomalous microstructure at the bottom of the specimen, as marked by the white rectangle in figure 2(a). The morphology of these anomalous microstructures (figure 2(c)) is very similar to the anomalous eutectic formed in the solidification of undercooled Ni-18.7at.%Sn eutectic alloy melt [13–17]. Figure 2(d) shows the transition zone between the normal microstructure with the coarse primary *α*-Ni dendrite and the fine regular eutectic in the interdendrite and the anomalous eutectic in the as-remelted specimen. It can be seen from figure 2(d) that the anomalous eutectic formed at the bottom of the laser remelted specimen, which consists of *α*-Ni particles in a Ni_3_Sn intermetallic compound matrix. According to the Ni–Sn equilibrium phase diagram (figure 1), the primary phase should be *α*-Ni solid solution phase during the solidification of the Ni-30 wt.% Sn hypoeutectic alloy. It is interesting to note that there is a primary Ni_3_Sn phase, as pointed out by the black arrow in figure 2(d), in the as-remelted Ni-30 wt.% Sn hypoeutectic alloy. It should be noted that the composition of the Ni-30 wt.% Sn alloy is close to the eutectic composition (Ni-18.7 at.% Sn). Consequently, both the Ni3Sn and *α*-Ni phases easily nucleate and grow in the Ni-30 wt.% Sn alloy melt. Wei *et al* [13] also demonstrated that both Ni_3_Sn and the *α*-Ni phase can nucleate primarily and independently during the solidification of the undercooled Ni-32.5 wt.% Sn eutectic alloy melt. Also observed is a thin Ni_3_Sn phase layer wrapping around the primary *α*-Ni dendritic arm, which looks like a halo structure. This suggests that the *α*-Ni phase should also be a good substrate for the nucleation and growth of the Ni_3_Sn phase.

### 3.2. Composition of anomalous eutectic

**Figure 3.**
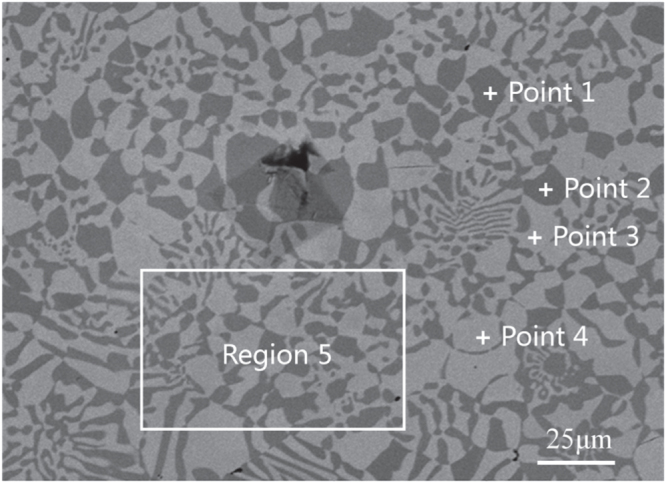
The anomalous eutectic zone analyzed by energy dispersive spectrometry (EDS).

Figure 3 shows the microstructure analyzed by scanning electron microscope with energy dispersive spectrometry. The white plus signs and the rectangular box are the positions and the region measured by EDS. The EDS results are listed in table 1. According to the Ni–Sn phase diagram in figure 1, the equilibrium concentration of Sn is 10.6 at.% in the *α*-Ni phase and 24.5 at.% in the Ni_3_Sn for the *α*-Ni + Ni_3_Sn eutectic. It is noted that Ni_3_Sn is not a stoichiometric compound, and its composition can change in a narrow range. Points 1 and 2 are located at *α*-Ni phases, points 3 and 4 are located at Ni_3_Sn compounds. But *α*-Ni phases are supersaturated with Sn element and Ni_3_Sn compounds contain less Sn element than the equilibrium concentration. As for the EDS result in region 5, it stands for the average composition of the *α*-Ni + Ni_3_Sn anomalous eutectic microstructure. The EDS result is consistent with the composition of the original ingot Ni-30 wt.% Sn. This suggests a significant change in the appearance, but little change in the composition, of the anomalous eutectic.

**Table 1.** The EDS results for the region and points shown in figure 2.

Place	Point 1	Point 2	Point 3	Point 4	Region 5
Composition (wt.% Sn)	21.2	20.2	39.6	39.1	30.9
Composition (at.% Sn)	11.8	11.2	24.5	24.1	18.1

### 3.3. Serial sectioning and EBSD analysis

**Figure 4.**
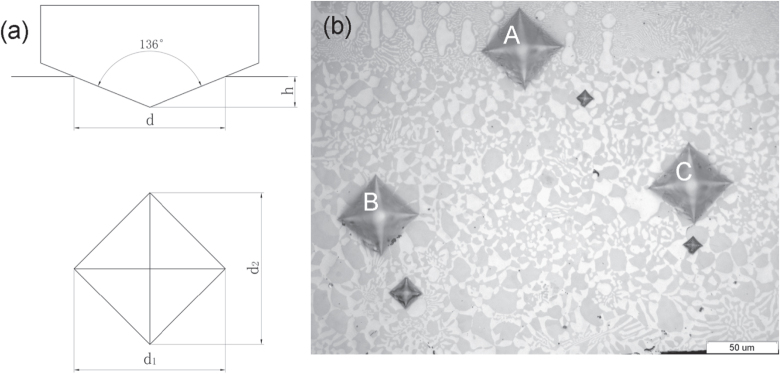
Vickers hardness indentation made by microhardness tester: (a) schematic diagram and geometric details of Vickers indentation [21]; (b) optical microscopy image showing the hardness indentations in the remelted specimen.

Figure 4 depicts the Vickers hardness indentations made by a microhardness tester. Geometric details of Vickers indentation and hardness indentations in the specimen are shown in figures 4(a) and (b), respectively. It should be pointed out that the image contrast differs in OM and SEM due to the different imaging principles. The Vickers indentations play two important roles in three-dimensional serial sectioning reconstruction. Firstly, they are used to trace the position in the arrangement of subsequent OM photos. Secondly, they can be used to calculate the thickness of the polished layer. The calculation formula of the indentation depth *h* (as shown in figure 4(a)) is expressed as follows:

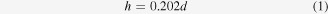
where *d* is the average distance of two diagonals of the indentation, (*d*_1_ + *d*_2_)/2. In the present paper, *d* represents the size of the Vickers indentation.

**Figure 5.**
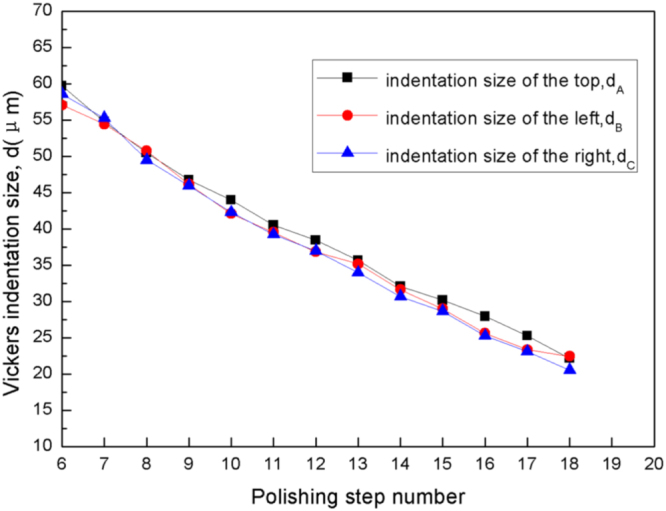
The variation in Vickers indentation size *d* with the number of polishing steps, as marked in figure 4.

Figure 5 presents the variation of Vickers indentation size *d* with the polished layer number with the number of polishing steps, each step taking 2 min. As can be seen in figure 5, the relationship between indentation size *d* and the polished layer number is nearly linear. So the average thickness Δ*h* of each layer removed by the fine polishing using the OP-S solution is 0.202 times the mean slope Δ*d* of the fitting lines in figure 5, i.e. Δ*h* = 0.202*Δ*d* = 0.59 *μ*m, when the polishing time for each layer is set to 2 min. This thickness of the polished layer is much less than the particle size of the anomalous eutectic, which can guarantee the precision of 3D reconstruction.

**Figure 6.**
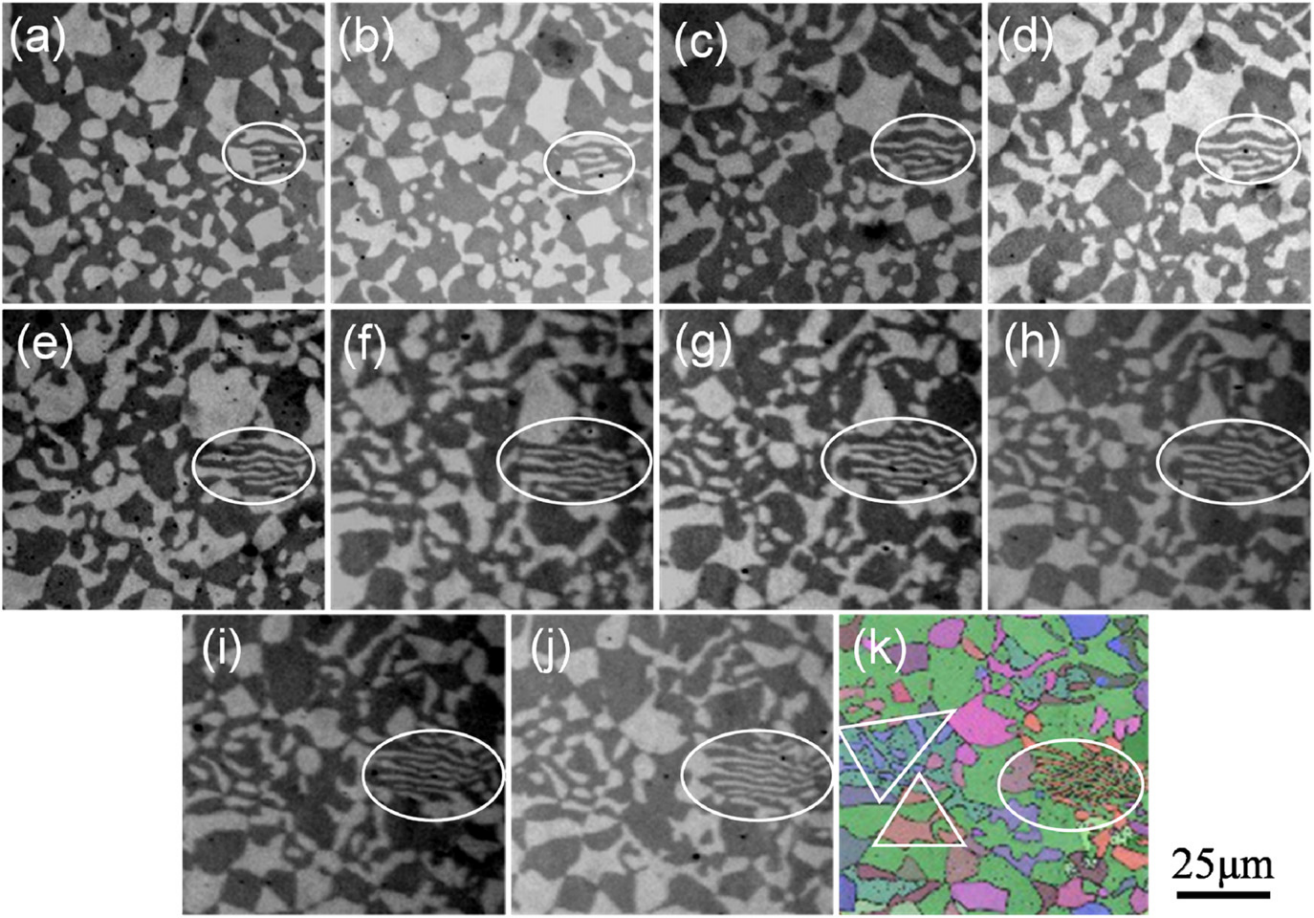
Anomalous eutectic morphology in the different polished layers and the EBSD pattern corresponding to the last layer: (a) 1st, (b) 5th, (c) 9th, (d) 13th, (e) 17th, (f) 21, (g) 25th, (h) 29th, (i) 33rd, (j) 37th layer (OM); (k) the EBSD relative Euler orientation map of the 37th layer.

Figure 6 illustrates the optical micrographs of the anomalous eutectic at different polished layers ((a)–(j)) and the EBSD pattern (k) corresponding to the last layer (j). In this series of images, the microstructure evolution of the Ni–Sn anomalous eutectic with the polished layers is presented. Although this sequence is not *in situ* during the evolution of the microstructure, they still show the complexity of the interconnections of the *α*-Ni + Ni_3_Sn anomalous eutectic microstructure. From the first layer, it seems that most of the region is composed of black Ni_3_Sn phase and white *α*-Ni particles, as shown in figure 6(a). There is a small volume fraction of lamella eutectic as marked in figure 6(a); the amount of lamellar eutectic increases in subsequent layers (figures 6(b)–(j)). At the 37th layer, the initial four short lamellae in the first layer have developed into a large area with the more wavy lamella in the lamella eutectic, as shown in figure 6(j).

The EBSD pattern collected in the 37th layer is shown in figure 6(k). All the Ni_3_Sn intermetallic compounds share the same crystallographic orientation since all of them are colored green in the EBSD relative Euler orientation map. *α*-Ni particles have various orientations. Li *et al* [23] also found similar results in the solidification of undercooled Ni–Sn melt.

From the optical microscope picture in figure 6(j), it can be seen that the two white *α*-Ni particles to the left of the ellipse region seem to be continuous with the white *α*-Ni lamella. However, combining this with the EBSD pattern (figure 6(k)), it can be deduced that they are not and have different orientations. Besides, it looks as though not all the black Ni_3_Sn particles continue in the optical microscope images. In some regions marked by white triangles in figure 6(k), the *α*-Ni particles have the same orientation, perhaps they are continuous in 3D space in these regions. To further verify the relative phase connectivity, it is necessary to reconstruct the 3D structure of the anomalous eutectic.

### 3.4. The 3D morphology of anomalous eutectic

**Figure 7.**
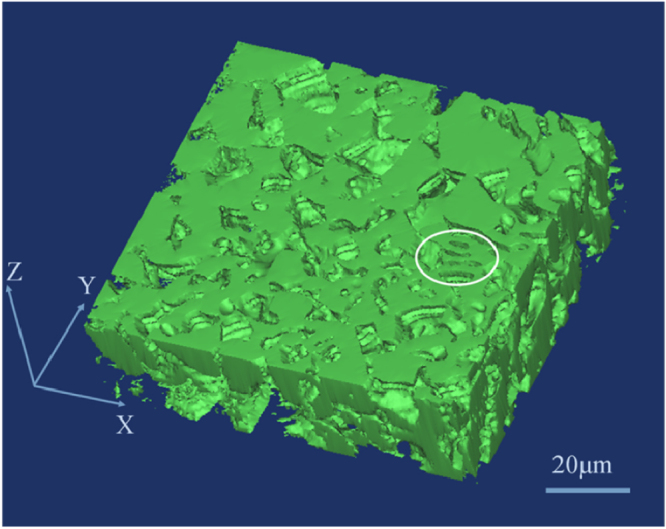
The 3D morphology of the Ni_3_Sn phase in the *α*-Ni + Ni_3_Sn anomalous eutectic. The entire reconstructed region is *X* = 97.1 *μ*m; *Y* = 90.2 *μ*m; *Z* = 21.8 *μ*m.

Figure 7 depicts the 3D morphology of the Ni_3_Sn phase in the *α*-Ni + Ni_3_Sn anomalous eutectic. As can be seen, the Ni_3_Sn phase presents a continuous interconnected network, which agrees with the EBSD result shown in figure 6(k). It should be pointed out that the lamella Ni_3_Sn phase marked by the white ellipse in figure 6(k) is also connected with the anomalous Ni_3_Sn matrix. Through the EBSD analysis in figure 6(k), it can be seen that the crystallographic orientation of the Ni_3_Sn lamella is the same as that of the polyhedral network Ni_3_Sn matrix. Kattamis and Flemings [11] first studied the microstructure of the undercooled Ni–Sn eutectic alloy. Through the successive polishing and examining of parallel sections for approximately 10 *μ*m, they concluded that both phases in the *α*-Ni + Ni_3_Sn anomalous eutectic were interconnected along a polyhedral network. However, in the present research, only the Ni_3_Sn phase is interconnected along a polyhedral network and the *α*-Ni phase is not interconnected.

**Figure 8.**
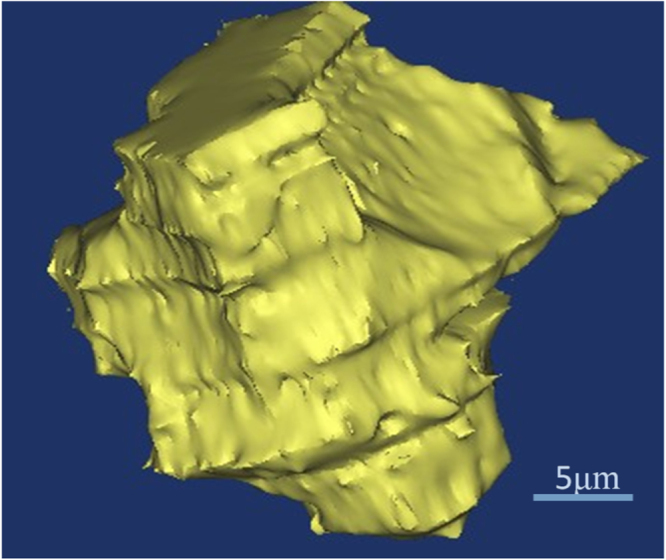
The 3D morphology of the *α*-Ni particle in the *α*-Ni + Ni_3_Sn anomalous eutectic.

From figure 6, it is interesting to note that there are two kinds of *α*-Ni particle in the anomalous eutectic. One seems to present a coupled growth with the adjacent Ni3Sn phase, which shows the consistent orientation with the other adjacent *α*-Ni particles (as marked by the white triangle in figure 6(k)). The other seems to present a decoupled growth with the adjacent Ni3Sn phase, which shows the random orientation with the other adjacent *α*-Ni particles. In the present work, the latter is reconstructed in three dimensions. Figure 8 shows the 3D morphology of a large *α*-Ni particle in the *α*-Ni + Ni_3_Sn anomalous eutectic. As can be seen, the surface of the *α*-Ni particle is irregular. Based on the Ni–Sn phase diagram in figure 1, as for the Ni-30 wt.% Sn hypoeutectic alloy, the primary *α*-Ni phase should precipitate first during the solidification process. It is speculated that these separate large *α*-Ni particles originated from the remelting of primary *α*-Ni dendrites. The solute concentration in the root of the primary dendrite is higher, and its corresponding melting point is lower, leading to the separation of dendritic arms which remelted into the separate particles.

**Figure 9.**
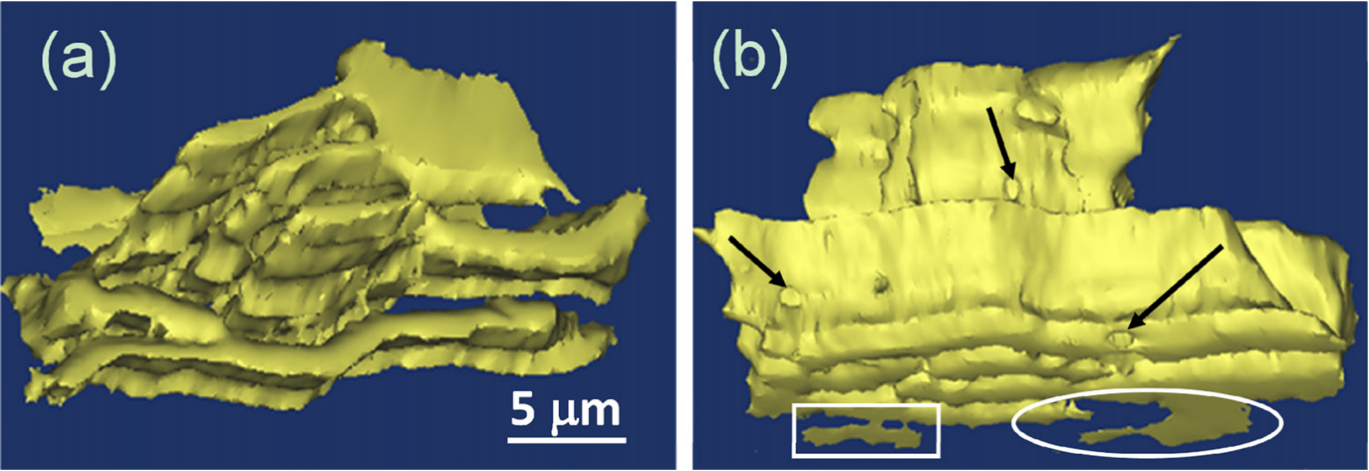
The 3D morphology of the lamella *α*-Ni phase in the *α*-Ni + Ni_3_Sn anomalous eutectic: (a)the top view of the lamella *α*-Ni phase; (b) the side view of the lamella *α*-Ni phase.

Figure 9 presents the 3D morphology of the lamella *α*-Ni phase marked by the white ellipse regions in figure 6. From the top view in figure 9(a), it can be seen that the lamella eutectic does not present the rigorous regular lamella and has a wavy microstructure. Akamatsu *et al* [24] have found that a zigzag instability will occur when the lamella spacing is above a critical spacing, which leads to a transition from the straight lamella to wavy lamella in the directional solidification of the transparent eutectic alloy. Trivedi *et al* [25] found that the recalescence effect in the unconstrained solidification will gives rise to nonsteady state growth conditions that could lead to the instabilities of lamellar eutectic growth by investigating the microstructure in the finely atomized droplets of Al-Si alloys. In the present work, the specimen was remelted thoroughly, so the solidification processing at the bottom of the molten pool was similar to the unconstrained solidification. As a result, the regular lamellar eutectic growth is not stable and the wavy eutectic microstructure appeared. Figure 9(b) shows the side view of the lamella *α*-Ni phase. At the bottom of the picture, there is a bump on the lateral surface of a lamella, as marked by the white ellipse, and a short discontinued lamellar is found in the area marked by the white rectangle. Walker *et al* [26] also found a similar phenomenon in the directional solidification of Al-33.2 wt.% Cu eutectic alloy. They indicated that there was a new 3D instability state for lamellar creation in Al-33.2 wt.% Cu eutectic alloy, through the observation of the successive transverse sections. They found that a perturbation to the eutectic lamella can grow and develop into a new lamella which propagates along the pre-existing ones after a sudden increase in the growth velocity from 1.25 to 5 *μ*m s^−1^.

Through the side view of the lamella *α*-Ni phase, holes are found in the positions marked with black arrows, as shown in figure 9(b). Zhao *et al* [27] also found holes in the primary Al_2_Cu phase in the 3D reconstruction of Al-40%Cu hypereutectic alloy. They suggested that if the latent heat of solidification cannot be released in time, it will lead to the remelting of the primary eutectic phase. This phenomenon has also been found in the 3D internal microstructural features of primary silicon crystals in a cast Al-Si base alloy [28]. In the present work, it can be deduced that the holes in the lamellar *α*-Ni phase may also mean the occurrence of remelting during solidification. Thus, the small *α*-Ni particles with the same orientation in some regions, marked by the white triangles in figure 6(k), may result from the remelting of the lamellar *α*-Ni phase. Recently, Wei *et al* [29] suggested that the remelting driven by chemical superheating dominates the anomalous eutectic formation regardless of whether the primary solid consists of a single phase or eutectic structure. The solid–liquid interface energy plays a role when the superheating induced remelting occurs. It may promote the breakup of the original structure at the post-recalescence solidification stage in the high undercooling solidification [29].

### 3.5. Formation condition for anomalous eutectic

In previous studies [11, 13, 23], the anomalous eutectic was thought to be the product of rapid solidification, while the lamellar eutectic formed under slow solidification conditions. Nevertheless, Wang *et al* [20] found that the microstructure in the remelted region was completely composed of a regular lamellar eutectic in laser surface remelting of Ni-33 wt.% Sn alloy when the laser scanning velocity was 20 mm s^−1^. But when the laser scanning velocity decreased to 0.1 mm s^−1^, *α*-Ni + Ni_3_Sn anomalous eutectic was found at the bottom of the remelted specimen. Meanwhile, Goetzinger *et al* [6] pointed out that when the cooling rate was above about 10^5^ K s^−1^, no transition from lamella to anomalous eutectic was observed for Ni_78.6_Si_21.4_ eutectic alloy solidified in drop-tube experiments. When the cooling rate was less than 10^5^ K s^−1^, the anomalous eutectic and lamellar eutectic coexisted in the specimen. They indicated that the tendency for a transition from the regular lamellar eutectic to the anomalous eutectic increases with the melt undercooling but decreases with the cooling rate. In the present work, it was found through serial sectional 3D reconstruction that the lamella eutectic coexists with the anomalous eutectic when the laser scanning speed is 0.1 mm s^−1^. This means that the anomalous eutectic formation is not only dependent on the change of the solidification rate.

Trivedi, Magnin and Kurz (TMK) [30] have developed a theory to describe the lamellar eutectic growth under rapid solidification. The TMK model can be used to describe the relationship among the lamellar spacing λ*E*, growth velocity *V* and the eutectic interface undercooling Δ*T*_E_ for the solidification of Ni–Sn eutectic alloy based on the Ni–Sn phase diagram (figure 1). Since the Ni-30 wt.% Sn hypoeutectic alloy specimen was remelted thoroughly and then resolidified, the moving velocity of the solid–liquid interface cannot be obtained from the laser scanning velocity. As a result, the lamellar spacing of the *α*-Ni + Ni_3_Sn lamellar eutectic was measured and the interface undercooling was estimated using the TMK model.

**Figure 10.**
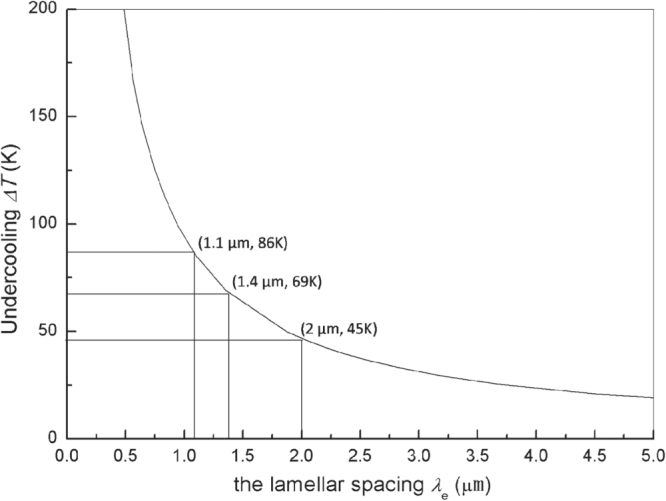
The variation of the undercooling Δ*T* with the lamellar spacing *λ*_e_ estimated using the TMK model.

According to the TMK model, Δ*T*_E_ can be solved by the following equations:

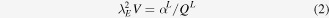


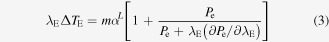
where

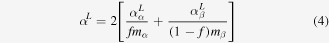


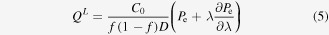


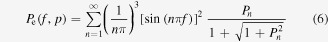


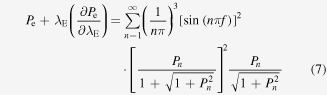


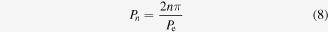
where *f* is the volume fraction of the *α* phase in the *α* + *β* eutectic, *m* is the velocity-dependent weighted liquidus slope, 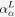 and 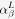 are the capillarity constants of the *α* and *β* phases, *P*_e_ is the solutal Peclet number for eutectic growth. The calculated variation curve of the undercooling with the *α*-Ni + Ni_3_Sn eutectic lamellar spacing is shown in figure 10. The thermal-physical parameters of the Ni–Sn system that were used are listed in table 2. The interface undercooling of the *α*-Ni + Ni_3_Sn lamellar eutectic solidification in the different positions of the specimen were also estimated according to the TMK model.

**Table 2.** The physical parameters of the Ni–Sn system used in the calculation (TMK model) [22, 31, 32].

Parameter	Symbol	Unit	Value
The eutectic temperature	*T*_E_	K	1412
The length of eutectic line	*C*_0_	at.	0.124
The eutectic composition	*C*_e_	at.	0.187
The volume fraction of *α*-Ni phase	*f*		0.32
Gibbs–Thompson coefficient of *α*-Ni	Γ*α*	Km	2.9 × 10^−7^
Gibbs–Thompson coefficient of Ni_3_Sn	Γ_*β*_	Km	2.1 × 10^−7^
Capillary constant of *α*-Ni	*a*_*L*_ (*α*)	J m^−2^	2.6×10^−7^
Capillary constant of Ni_3_Sn	*a*_*L*_ (*β*)	J m^−2^	1.1 × 10^−7^
The liquid slope of *α*-Ni	*mα*	K/at.	−2100
The liquid slope of Ni_3_Sn	*m*_*β*_	K/at.	3700
Equilibrium distribution coefficient of *α*-Ni	*kα*		0.57
Equilibrium distribution coefficient of Ni_3_Sn	*k*_*β*_		0.32
Equilibrium diffusion coefficient in liquid	*D*_0_	m^2^ s^−1^	2.8 × 10^−8^
Gas constant	*R*	J (K mol)^−1^	8.314
Activation energy	*Q*	J mol^−1^	21 197

At the bottom of the specimen, the lamellar spacing *λ*_e_ of the *α*-Ni + Ni_3_Sn lamellar eutectic mixed with the *α*-Ni + Ni_3_Sn anomalous eutectic (shown in figure 6) is ∼2 *μ*m and the estimated undercooling Δ*T* is ∼45 K. In the middle of the specimen, the lamellar spacing (shown in figure 2(d)) is ∼1.4 *μ*m and the estimated undercooling Δ*T* is ∼69 K. At the top of the specimen, the lamellar spacing (shown in figure 2(b)) is ∼1.1 *μ*m and the estimated undercooling Δ*T* is ∼86 K. The estimated undercoolings in the three regions of the specimen all exceed the critical undercooling reported by the references [15, 16], but the anomalous eutectic is not found at the top or in the middle of the specimen. That means that the critical undercooling could not be the sufficient condition for the formation of the anomalous eutectic.

In the previous literature [11–15], it was believed that the two phases of a eutectic cannot maintain coupled growth when the growth velocity exceeds the growth velocity threshold for coupled eutectic growth, and then the two phases grow to decouple into the anomalous eutectic. But in the present work it is found that *α*-Ni + Ni_3_Sn anomalous eutectic mixed with the lamella eutectic. In addition, from the EBSD analysis, the orientation of the lamella Ni_3_Sn phase is the same as the polyhedral network Ni_3_Sn matrix in the anomalous eutectic. The orientation of *α*-Ni phase in the lamella is consistent although it is different from the orientations of surrounding *α*-Ni particles. In particular, the *α*-Ni particles do not present a complete random misorientation. It can be found that the crystallographic orientations of some *α*-Ni particles (marked by white triangles in figure 6(k)) in the *α*-Ni + Ni_3_Sn anomalous eutectic are consistent. This also means both the coupled and decoupled growth of the eutectic *α*-Ni and Ni_3_Sn phases can form the anomalous eutectic.

It should be pointed out that in the previous works [13, 15, 23] the anomalous eutectics were generally surrounded by lamellar eutectic structures in the solidification of the undercooled eutectic melt. Wei *et al* [13] pointed out that the Ni–Sn anomalous eutectic forms at the initial stage of the rapid solidification of high undercooling alloy melt and then the lamellar eutectic forms in the residual alloy melt when the melt undercooling reaches a certain value. Li *et al* [23] deduced that the dissipation of latent heat from *α*-Ni and Ni_3_Sn solidification can lead to the fragmentation of *α*-Ni within the central part of the *α*-Ni + Ni_3_Sn anomalous eutectic colony. Meanwhile, the dissipation of latent heat can increase the temperature of the residual liquid ahead of the growing interface and lower the interface undercooling. It thus enables the regular *α*-Ni + Ni_3_Sn eutectic lamellae to develop from the periphery of the *α*-Ni + Ni_3_Sn anomalous eutectic grain. However, in the present work, the lamella eutectic is surrounded by anomalous eutectics, which is different from the results in the solidification of the undercooled eutectic melt. Through the reconstruction 3D morphology, it can be deduced that the remelting happened in the lamella *α*-Ni phase based on the observed holes at the surface of the lamella eutectic. Combined with the EBSD result, the small *α*-Ni particles with the same orientation in some regions may result from the remelting of the lamellar *α*-Ni phase. This indicates that the lamella eutectic may perhaps form prior to the formation of the anomalous eutectic.

**Figure 11.**
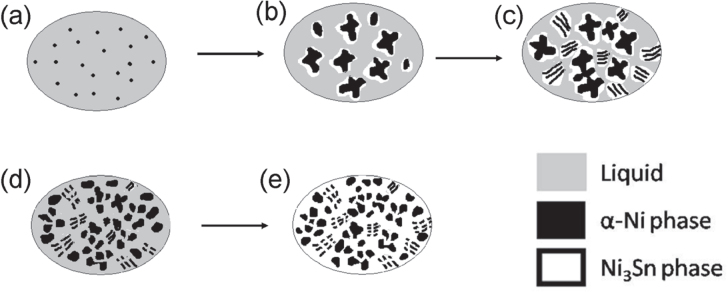
Schematic of the formation of the *α*-Ni + Ni_3_Sn anomalous eutectic at the bottom of laser remelted Ni-30 wt.% Sn hypoeutectic alloy.

According to the points mentioned above, it can be deduced that the possible formation mechanism and schematic illustration (figure 11) for a *α*-Ni + Ni_3_Sn anomalous eutectic at the bottom of a laser remelted Ni-30 wt.% Sn hypoeutectic specimen should be as follows.

According to the Ni–Sn phase diagram in figure 1, when the Ni-30 wt.% Sn alloy melt is cooling, the primary *α*-Ni phase should nucleate first during the solidification process for the Ni-30 wt.% Sn hypoeutectic when the undercooling Δ*T* is ∼33 K (figure 11(a)). As the *α*-Ni phase grows up, the solute element Sn will be rich in the liquid around the *α*-Ni phase, which leads the nucleation driving force of the Ni_3_Sn phase to increase. On considering that the *α*-Ni phase is also a suitable nucleation substrate of the Ni_3_Sn phase, the Ni_3_Sn phase nucleates easily on the *α*-Ni phase with the growth of the *α*-Ni phase (figure 11(b)). With the further solidification of both the *α*-Ni phase and the Ni_3_Sn phase, the local residual liquid around these two phases will tend to the Ni-32.5 wt.% Sn eutectic composition due to the solute segregation, which leads both the *α*-Ni and the Ni_3_Sn phase to present a coupled growth behavior and form a lamella eutectic (figure 11(c)). It can be seen from figure 1 that the melting point of the Ni_3_Sn phase is ∼1412 K and the melting point of the *α*-Ni phase is ∼1445 K. The former is lower than the latter one. The latent heat accumulated in the process of solidification can make the *α*-Ni phase remelt locally and make the Ni_3_Sn phase remelt completely (figure 11(d)). Finally, when the Ni-30 wt.% Sn alloy is cooled to room temperature*, α*-Ni + Ni_3_Sn anomalous eutectics with different scales are formed (figure 11(e)).

## 4. Conclusions

Ni-30 wt.% Sn hypoeutectic alloy was remelted using a laser and *α*-Ni + Ni_3_Sn anomalous eutectic was found at the bottom of the specimen. The three-dimensional morphologies of the *α*-Ni phase and the Ni_3_Sn phase in the anomalous eutectic were reconstructed using serial sectional technology. Combined with EDS, EBSD detections and the TMK model, the formation of the anomalous eutectic was analyzed. The main findings of this study can be summarized as follows.

1. The *α*-Ni + Ni_3_Sn anomalous eutectic was found at the bottom of the molten pool when the specimen of Ni-30 wt.% Sn hypoeutectic alloy was remelted thoroughly. The anomalous eutectic is composed of the coarse *α*-Ni particles and the Ni_3_Sn intermetallic compound matrix. The coarse primary *α*-Ni dendrite and the fine regular *α*-Ni + Ni_3_Sn lamellar and rod eutectic in the interdendrite exist in most regions of the remelted specimen.
2. The average composition of the *α*-Ni + Ni_3_Sn anomalous eutectic is near Ni-30 wt.% Sn, which is close to the original composition. This means that the *α*-Ni + Ni_3_Sn anomalous eutectic undergoes a significant change in appearance but little change in composition. The granular *α*-Ni phase is supersaturated with the Sn element in the anomalous eutectic, which is in accordance with the feature of supersaturated solid solution under the rapid solidification condition.
3. The 3D morphology of the *α*-Ni and Ni_3_Sn phases in the anomalous eutectic are obtained by serial sectioning reconstruction technology. Most of the *α*-Ni phases are the discontinued particles and the Ni_3_Sn phase is a continuous interconnected network structure, which is in agreement with the EBSD analysis.
4. The wavy lamella eutectic was mixed with the anomalous eutectic, and the orientation of the lamella Ni_3_Sn phase is the same as that of the Ni_3_Sn polyhedral network matrix in the anomalous eutectic. The orientation of the *α*-Ni phase in the lamella eutectic is consistent, but it is different from the orientations of the surrounding *α*-Ni particles. Besides, *α*-Ni particles do not present a completely random misorientation. This indicates that both coupled and decoupled growth of the *α*-Ni and Ni_3_Sn phases can generate the anomalous eutectic structure.
5. The TMK model was used to estimate the undercoolings with the eutectic lamellar spacing from the bottom to the top of the specimen. When the undercoolings exceed the critical undercooling reported by a previous researcher, no anomalous eutectic appears at the top or middle of the specimen. These results mean that critical undercooling could not be the sufficient condition for the formation of an anomalous eutectic.
